# Spike-timing-dependent plasticity rewards synchrony rather than causality

**DOI:** 10.1093/cercor/bhac050

**Published:** 2022-02-24

**Authors:** Margarita Anisimova, Bas van Bommel, Rui Wang, Marina Mikhaylova, Jörn Simon Wiegert, Thomas G Oertner, Christine E Gee

**Affiliations:** Center for Molecular Neurobiology Hamburg, University Medical Center Hamburg-Eppendorf, Falkenried 94, D-20251 Hamburg, Germany; Center for Molecular Neurobiology Hamburg, University Medical Center Hamburg-Eppendorf, Falkenried 94, D-20251 Hamburg, Germany; Institute for Chemistry and Biochemistry, Feie Universität Berlin, Berlin, Germany; Center for Molecular Neurobiology Hamburg, University Medical Center Hamburg-Eppendorf, Falkenried 94, D-20251 Hamburg, Germany; Center for Molecular Neurobiology Hamburg, University Medical Center Hamburg-Eppendorf, Falkenried 94, D-20251 Hamburg, Germany; Institute of Biology, Humboldt-Universität zu Berlin, Berlin, Germany; Center for Molecular Neurobiology Hamburg, University Medical Center Hamburg-Eppendorf, Falkenried 94, D-20251 Hamburg, Germany; Center for Molecular Neurobiology Hamburg, University Medical Center Hamburg-Eppendorf, Falkenried 94, D-20251 Hamburg, Germany; Center for Molecular Neurobiology Hamburg, University Medical Center Hamburg-Eppendorf, Falkenried 94, D-20251 Hamburg, Germany

**Keywords:** hippocampus, LTP, optogenetics, rat, synaptic plasticity

## Abstract

Spike-timing-dependent plasticity (STDP) is a candidate mechanism for information storage in the brain, but the whole-cell recordings required for the experimental induction of STDP are typically limited to 1 h. This mismatch of time scales is a long-standing weakness in synaptic theories of memory. Here we use spectrally separated optogenetic stimulation to fire precisely timed action potentials (spikes) in CA3 and CA1 pyramidal cells. Twenty minutes after optogenetic induction of STDP (oSTDP), we observed timing-dependent depression (tLTD) and timing-dependent potentiation (tLTP), depending on the sequence of spiking. As oSTDP does not require electrodes, we could also assess the strength of these paired connections three days later. At this late time point, late tLTP was observed for both causal (CA3 before CA1) and anticausal (CA1 before CA3) timing, but not for asynchronous activity patterns (Δ*t* = 50 ms). Blocking activity after induction of oSTDP prevented stable potentiation. Our results confirm that neurons wire together if they fire together, but suggest that synaptic depression after anticausal activation (tLTD) is a transient phenomenon.

## Introduction

Synapses change their strength in response to synchronized activity in pre- and post-synaptic neurons. This fascinating property, commonly referred to as spike-timing-dependent plasticity (STDP), has inspired numerous computational models of memory formation (Gerstner et al. 1996; Song et al. 2000; Masquelier et al. 2009; Costa et al. 2015). Indeed, STDP-like learning rules are now widely used for unsupervised learning, have been implemented on neuromorphic hardware and in robotics (Bing et al. 2020; Kheradpisheh et al. 2018; Serrano-Gotarredona et al. 2013). Canonical STDP bidirectionally changes synaptic strength depending on the order of pre- and post-synaptic activity. Timing-dependent long-term potentiation (tLTP) occurs when excitatory synaptic potentials repeatedly precede action potentials, i.e. causal or Hebbian pairing. In contrast, timing-dependent long-term depression (tLTD) occurs when this order is reversed, i.e. anticausal pairing (Markram 1997; Bi and Poo 1998; Debanne et al. 1998). Depending on the synapse in question, the exact spiking pattern and the presence or absence of neuromodulators, different STDP rules have been described (Foncelle et al. 2018; Brzosko et al. 2019). Typically, STDP experiments follow changes in synaptic strength for 20–60 min after induction. This time limitation arises from the control invasive nature of intracellular recordings, which are necessary to the precise timing of postsynaptic spikes. Memories last much longer. Our aim was therefore to extend the post-STDP observation time from hours to several days and examine the late effects on synaptic strength.

Channelrhodopsins are light-activated ion channels, and since the discovery of channelrhodopsin-2 (Nagel et al. 2003) have been widely used to stimulate neurons in vitro and in vivo (e.g. Boyden et al. 2005; Adamantidis et al. 2007; Huber et al. 2008). In recent years, channelrhodopsin variants with different kinetics and spectral sensitivities have been discovered or engineered (e.g. Hochbaum et al. 2014; Klapoetke et al. 2014). Here, we present a 2-color method to evoke precisely timed action potentials in defined subsets of CA3 and CA1 pyramidal neurons for optogenetic induction of STDP (oSTDP). By combining the opsin CheRiff and less sensitive ChrimsonR, we could independently drive precisely timed action potentials in CA1 and CA3 pyramidal neurons using 1.3 mW mm^−2^ 405 nm and 8 mW mm^−2^ 625 nm light pulses, respectively. As expected, we found that during short-term patch-clamp recordings (20 min after induction), oSTDP resulted in tLTP after causal (single presynaptic spike before postsynaptic burst) pairing and tLTD after anticausal (postsynaptic burst before single presynaptic spike) pairing, a classical asymmetric plasticity window. However, 3 days after oSTDP, by comparing synaptic responses of paired neurons with those of their nontransfected (NT) neighbors, the window became relatively symmetric, comprised of tLTP at short (±10 ms) delays and no change with longer (±50 ms) delays (i.e. phase-shifted activity). Late tLTP depended on pairing frequency (at 5 Hz but not at 0.1 Hz) and activation of NMDA-type glutamate receptors. We also observed that the appearance of late tLTP after anticausal pairing requires ongoing spontaneous activity in the network. Our findings reveal that short-term recordings of STDP cannot simply be extrapolated to predict synaptic strength over behaviorally relevant time scales. Especially, the frequently reported tLTD might not be “long-term” after all.

## Materials and methods

### Rat organotypic hippocampal slice cultures preparation and transfection

Wistar rats were housed and bred at the University Medical Center Hamburg-Eppendorf (UKE) animal facility and sacrificed according to German Law (Tierschutzgesetz der Bundesrepublik Deutschland, TierSchG) with approval from the Behörde für Justiz und Verbraucherschutz (BJV)-Lebensmittelsicherheit und Veterinärwesen Hamburg, and the animal care committee of the UKE. The procedure for preparing organotypic cultures was modified from Stoppini et al. (Stoppini et al. 1991), using media without antibiotics (Gee et al. 2017). Cultures were transduced at 10–11 days in vitro (DIV) with local injection (Wiegert et al. 2017a) into CA3 of AAV2.Rh10-syn-ChrimsonR-tdTomato 7.22 × 10^13^ vg mL^−1^ (packaged at the UKE Vector Facility, plasmid a gift from Edward Boyden). On the same day, CA1 neurons were transfected with pAAV-hsyn-CheRiff-eGFP (0.5 ng μL^−1^) and pCI-syn-mKate2 (10 ng μL^−1^) using single cell electroporation (Wiegert et al. 2017b). After opsins were expressing, great care was taken to protect the cultures from wavelengths of light below 560 nm to prevent unintentional coactivation of ChrimsonR and CheRiff-expressing neurons. Handling and normal medium changes (2/3, twice per week) were performed under dim yellow light (Osram LUMILUX CHIP control T8).

### Experimental design

**Fig. 1:**
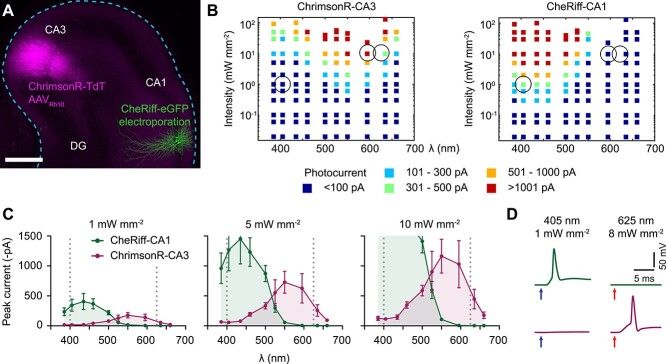
Selecting wavelength and intensity for independent spiking of CheRiff-CA1 and ChrimsonR-CA3 neurons. (A) Confocal image (maximum intensity projection) of an organotypic hippocampal slice culture (DIV 21) taken 7 days after transducing ~30 CA3 neurons with AAV_Rh10_-syn-ChrimsonR-tdTomato (magenta) and electroporating 4 CA1 neurons with pAAV-syn-CheRiff-eGFP (green). Scale bar 500 μm. (B) Color-coded photocurrent amplitude in response to 1 ms light flashes of varying intensity and wavelength of ChrimsonR-CA3 (left) and CheRiff-CA1 neurons (right). Circles indicate parameters used in plasticity experiments. *n* = 4 recordings per point, from 11 ChrimsonR-CA3 and 7 CheRiff-CA1 neurons. (C) Photocurrent amplitude vs wavelength from the data in (B) at intensities of 1, 5, and 10 mW mm^−2^. Regions of overlap will depolarize both CheRiff- and ChrimsonR-expressing neurons. At 10 mW mm^−2^, the maximum current recorded from CheRiff-CA1 neurons was −2100 ± 500 pA at 435 nm (mean ± SEM). Dotted lines indicate the optimal wavelengths to independently activate ChrimsonR-CA3 and CheRiff-CA1 neurons. Mean ± SEM, *n* = 4 measurements per point. (D) Example membrane responses of a CheRiff-CA1 (upper traces) and a ChrimsonR-CA3 (lower traces) neuron to 2 ms light flashes at 405 nm (1 mW mm^−2^, blue arrows) and 625 nm (8 mW mm^−2^, red arrows). Note the CheRiff-CA1 neuron fires a single action potential and the ChrimsonR-CA3 neuron only slightly depolarizes in response to 405 nm light, whereas the ChrimsonR-CA3 neuron fires one action potential in response to 625 nm and there is no response in the CheRiff-CA1 neuron. Note that in these experiments synaptic transmission was blocked.

**Fig. 2:**
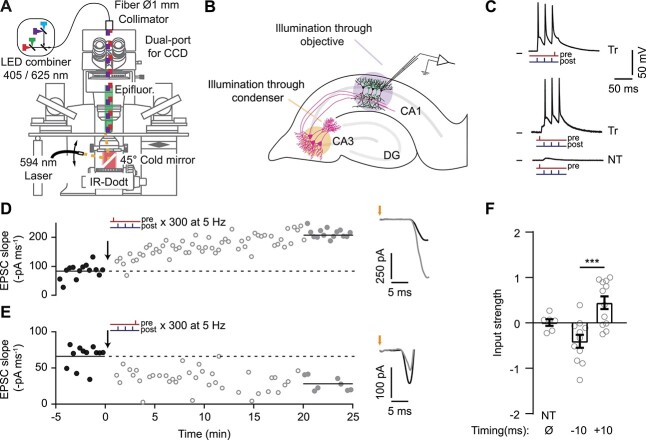
Optogenetic induction of oSTDP produces or timing-dependent tLTP and tLTD. (A) Diagram of the electrophysiological recording setup for on-axis CA1 stimulation through the objective and off-axis CA3 stimulation through the condenser. (B) Experimental configuration with patch-electrode in CA1. (C) Current-clamp recordings from CA1 neurons during oSTDP induction. Top, a CheRiff-transfected (Tr) CA1 neuron during anticausal pairing (−10 ms: 3 violet (405 nm) flashes at 50 Hz and 1 red (625 nm) flash 8 ms after, repeated 300x at 5 Hz). Middle, a CheRiff-transfected CA1 neuron during causal pairing (+10 ms: 1 red flash and 3 violet flashes at 50 Hz 12 ms after). Bottom, a NT CA1 neuron during causal pairing. Black ticks at left indicate −70 mV. (D) Left, example causal pairing experiment from one CheRiff-CA1 neuron. EPSCs were evoked by light stimulation of ChrimsonR-CA3 neurons before (black points) and after (gray points) causal pairing at *t* = 0 (black arrow). The filled points were significantly different, *P* < 0.0001, Kolmogorov–Smirnov. Right, averaged EPSCs from the filled points, orange arrow indicates stimulation of ChrimsonR-CA3 neurons. (E) As in panel (D), but after anticausal stimulation (black arrow). The filled gray points were significantly different to the baseline, *P* = 0.0003, Kolmogorov–Smirnov. (F) Normalized change in EPSC slope 20–25 min after oSTDP induction as in panels (D) (+10, *n* = 12) and E (−10, *n* = 11). NT, nontransfected neurons from slices subjected to causal pairing stimulation (*n* = 6). ^***^P = 0.0003, ANOVA-Sidak. Mean ± SEM

Except for data presented in Figures 1 and 2, all experiments and analyses were performed blind to the in-incubator optical stimulation or any drug treatments. Unblinding only occurred after initial analyses and any decision to include/exclude recordings had been taken. To ensure blinding, we always interspersed different treatment conditions with control (nonpaired) slices (Fig. 4D) and ±10 ms groups (Fig. 4E).

### CheRiff and ChrimsonR characterization

For characterizing the responses to multiple wavelengths, we used a pE-4000 CoolLED (CoolLED Ltd, s/n CP0180) with an additional filter for 550 nm (555 ± 20 nm). Light intensities in the specimen plane were measured using a 918D-ST-UV sensor and a 1936R power meter (Newport). Light flashes (1 ms, low-to-high intensity) were delivered every 20 s with 1 min between wavelength sequences. Whole cell currents were recorded (see electrophysiology below) with synaptic transmission and action potentials blocked by CPPene (10 μM, Tocris bioscience; 1265), NBQX (10 μM, Tocris bioscience; 1044), picrotoxin (100 μM, Sigma; P1675-1G), and tetrodotoxin (TTX, 1 μM, HelloBio; HB1035). We recorded from 7 CA1 (CheRiff) and 11 CA3 (ChrimsonR) neurons to obtain *n* = 4 responses at each wavelength/intensity combination. Recordings were performed in the recording medium described below (see optogenetic induction of STDP (oSTDP) during whole-cell recordings).

### Combined electrophysiology and optogenetic stimulation

The setup is based on an Olympus BX61WI microscope fitted with Dodt contrast, epifluorescence and a CCD-camera (DMK23U274, The Imaging Source) on a dual camera port (see Fig. 2A). For light stimulation through the objective, a LED combiner (Mightex Systems, wavelengths 625, 530, 470, 405 nm) was coupled via a 1 mm multimode fiber and a collimator (Thorlabs) mounted on one of the camera ports. A 40x water immersion objective (Plan-Apochromat 1.0 NA DIC VIS-IR, Zeiss, illuminated field ø 557 μm) or a 10x water immersion objective (UMPlanFL 0.30 NA W, Olympus, illuminated field ø 2.65 mm) were used. The light intensities through the objectives were measured in the specimen plane using a calibrated power meter (LaserCheck, Coherent or 918D-ST-UV sensor/1936R power meter, Newport). To locally activate the CA3 neurons, the orange (594) laser beam of an Omicron Light Hub was coupled into an optical fiber fitted with a collimator (Thorlabs) mounted on a lockable swing arm. This construction allowed us to point the laser beam at different angles through the center of the back aperture of the oil immersion condenser (NA 1.4, Olympus) to locally illuminate neurons in CA3 away (~1.5 mm) from the optical axis, with high intensity and little light scattering (Fig. 2A and B). After final laser positioning, the stage was not moved and CA1 neurons within the single field of view were recorded from.

Whole-cell patch-clamp recordings were performed using an Axopatch 200B (Axon Instruments, Inc.) amplifier, National Instruments A/D boards and Ephus software (Suter et al. 2010). EPSCs (Fig. 4) and cellular parameters (Supplementary Fig. S1) were measured at 30 °C (in-line heater, Warner Instruments) in artificial cerebral spinal fluid containing: NaCl (119 mM, Sigma; S5886-500G), NaHCO_3_ (26.2 mM, Sigma; S5761-500G), D-glucose (11 mM, Sigma; G7528-250G), KCl (2.5 mM, Fluka; 60,121-1 L), NaH_2_PO_4_ (1 mM, Sigma; S5011-100G), MgCl_2_ (4 mM, Fluka; 63,020-1 L), CaCl_2_ (4 mM, Honeywell; 21,114-1 L), pH 7.4, ~310 mOsm kg^−1^, saturated with humidified 95% O_2_/5% CO_2_. The patch electrodes (3–4 MΩ, World Precision Instruments; 1B150F-3) were filled with intracellular solution containing: K-gluconate (135 mM, Sigma; G4500-100G), EGTA (0.2 mM, Sigma-Aldrich; E0396-10G), HEPES (10 mM, Sigma; H4034-100G), MgCl_2_ (4 mM, Fluka; 63,020-1 L), Na_2_-ATP (4 mM, Aldrich; A26209-1G), Na-GTP (0.4 mM, Sigma; G8877-100MG), Na_2_-phosphocreatine (10 mM, Sigma; P7936-1G), ascorbate (3 mM, Sigma; A5960-100G), pH 7.2, 295 mOsm kg^−1^. The liquid junction potential was measured (−14.1 to −14.4 mV) and corrected. CA1 pyramidal neurons were patched under the 40x water immersion objective and voltage-clamped at −70 mV (LJP corrected). A test pulse (−5 mV, 200 ms) was applied at the start of every sweep. The series resistance (Rs) was less than 20 MΩ, did not change more than 30% and was not compensated in voltage clamp. In current clamp, we used bridge balance compensation.

### Optogenetic induction of spike-timing dependent plasticity during whole-cell recordings

About 8–11 days after virus injection and electroporation of ChrimsonR and CheRiff (18–22 DIV), the slices were transferred to the electrophysiology setup and perfused with slice culture medium containing (for 500 mL): 494 mL Minimal Essential Medium (Sigma M7278), 1 mM L-glutamine (Gibco 25030-024), 0.01 mg mL^−1^ insulin (Sigma I6634), 1.45 mL 5 M NaCl (S5150 Sigma), 2 mM MgSO_4_ (Fluka 63126), 1.44 mM CaCl_2_ (Fluka 21114), 0.00125% ascorbic acid (Fluka 11140), 13 mM D-glucose (Fluka 49152), supplemented with D-serine (30 μM, Tocris Bioscience; 0226), and diluted slightly to give ~308 mOsm kg^−1^ (33 °C, saturated with humidified 95% O_2_/5% CO_2_). The LJP was corrected (−15.3 mV). The medium (50 mL) was recycled during the experiment and changed every 4 h or every 4 slices, whichever came first. With the 594 nm laser positioned to stimulate ChrimsonR-CA3 neurons, a CA1 neuron was patched and EPSCs were recorded (every 20s) at −65.3 mV (LJP corrected) where inhibitory currents were clearly outward. The laser intensity was set to evoke EPSCs of approximately −100 pA (range −50 to −200 pA). Baseline EPSCs were recorded for up to 5 min after break-in. The 10x objective was carefully moved into position to maximize the area of the slice illuminated and the amplifier was switched to current clamp to allow spiking of the CA1 neuron during paired optogenetic stimulation. To induce plasticity, we used 300 spike-burst pairings (Nevian and Sakmann 2006; Wittenberg and Wang 2006; Frey et al. 2009). Stimulation of ChrimsonR-CA3 (594 nm laser at baseline intensity plus red LED stimulation through the objective; 625 nm, 2 mW mm^−2^, 2 ms, 5 Hz) was paired with bursts of 3 violet LED stimuli through the objective (405 nm, 1 mW mm^−2^, 2 ms, 50 Hz) to activate CheRiff-CA1 neurons. The additional red LED stimulation was added to more closely simulate the wide-field illumination of both CA3 and CA1 neurons when stimulated in the incubator. After pairing CA3 and CA1 stimulation, EPSCs (0.05 Hz orange laser flashes) were recorded for an additional 25 min. Pairing was always completed within 10 min of break-in.

### In-incubator oSTDP, read-out, and analysis

We waited 7–10 days (17–21 DIV) to ensure stable expression of ChrimsonR and CheRiff. The closed petri dish containing the centered slice culture was placed in an LED illumination tower constructed from 30 mm optical bench parts (Thorlabs) situated inside a dedicated incubator. The tower contained an injection-molded reflector (Roithner LaserTechnik, 10034) to collimate the 625 nm LED (Cree XP-E red) and an aspheric condenser lens (Thorlabs ACL2520U-A) to collimate the 405 nm LED (Roschwege Star-UV405-03-00-00). The LEDs were powered and controlled from outside the incubator by a 2-channel Grass S8800 stimulator, 2 constant-current drivers (RECOM RCD-24-1.20) and a timer. The LED power was adjusted to give 1 mW mm^−2^ for 405 nm and 8 mW mm^−2^ for 625 nm inside the petri dish in the specimen plane. “Non-paired” cultures were handled identically, but no light stimulation was given. After pairing, each petri dish was marked with a letter code and date/time of pairing. Where indicated, CPPene (1 μM, Fig. 4H) was added to the slice culture medium the day before or (Fig. 4I) 3–4 h after pairing the medium was changed to medium containing TTX (1 μM) to stop all spiking. Two days later, the slices were washed with fresh medium and returned to the incubator until the following day when they were recorded from.

Three days or 1 h after in-incubator stimulation, the slices were transferred by a different blinded investigator to the electrophysiology setup with on/off axis stimulation and perfused with ACSF at 30 °C as described above. Whole-cell patch-clamp recordings were sequentially made from 3 to 4 NT-CA1 pyramidal neurons and 1 to 5 CheRiff-CA1 pyramidal neurons in a pseudorandom order without moving the stage to ensure constant CA3 stimulation (holding potential −70 mV to ensure separation between EPSCs and IPSCs). While still in cell-attached mode, CA1 neurons were illuminated with a single 405 nm light flash (1 ms, 1 mW mm^−2^). None of the 255 NT-CA1 neurons spiked. Of 224 fluorescent CheRiff-CA1 neurons, 96% spiked in response to the single violet flash, 202 fired a single spike (90.2%) and 13 fired 2–3 spikes (5.8%). The recordings were immediately discontinued from the remaining 9 neurons that did not spike (4%). While collecting these data, spontaneous break-in occurred in 10 of the NT-CA1 neurons before the violet light flash. None showed a photocurrent or an EPSC that would have indicated recurrent CA1–CA1 connections were prevalent.

After break-in, ChrimsonR-CA3 neurons were stimulated through the condenser (594 nm, 20 s interval) to evoke EPSCs in the CA1 neurons. The EPSC onset was typically 6–8 ms after the stimulation. If the delay to the EPSC onset was 13 ms or longer, connections were assumed to be polysynaptic and not analyzed. Ten EPSCs were recorded at 3 laser intensities from each CA1 neuron (when spontaneous events obscured the EPSCs, extra sweeps were collected). The middle intensity was set to evoke an EPSC of about −100 to −300 pA in the first recording from a given slice. The same 3 intensities were used for all neurons in a given slice, but only 1 intensity was used for analysis. The selected intensity evoked the most similar EPSC slopes that were at least 20 pA in three NT cells (usually the lowest laser intensity).

At the end of each recording, we injected a series of current steps to assess cellular excitability. Cells without pyramidal-like firing patterns (i.e. high AP frequency, large amplitude after-hyperpolarization) were eliminated from the analysis. Individual sweeps with spontaneous EPSCs/IPSCs preceding the stimulation by less than 10 ms were not analyzed. If fewer than 4 sweeps remained at the selected laser intensity, the recording was discarded. A custom MATLAB routine averaged the EPSCs, detected the peak and the 20–60% slope (Fig. 4C). If the time to peak varied by more than 5 ms between neurons, we suspected a mixture of mono- and poly-synaptic responses and the slice was excluded.

### Immunohistochemistry

An hour after stimulation, the slices were fixed (30 min, ice-cold 4% paraformaldehyde in PBS). The slices were washed in PBS (3 x 10 min), blocked (2 h 5% donor horse serum, 0.3% TritonTM X-100 in PBS at room temperature) and then incubated overnight with the primary antibody (4 °C, rabbit anti-cFos, Santa Cruz Biotechnology, Inc., sc-52, 1:500) in carrier solution (1% Bovine serum albumin, 0.3% TritonTM X-100 in PBS). On the next day, the slices were washed 3 times in PBS and incubated at room temperature for 2 h with a secondary antibody (Alexa Fluor 568 goat anti-rabbit, Life Technologies, A11011) at 1:1000 in carrier solution (same as above) and afterwards washed again (3 x 10 min) in PBS and mounted with a Shandon Immu-Mount (Thermo Scientific; 9990402).

### Confocal microscopy

An Olympus F1000 confocal microscope with 20x objective (UPLSAPO 0.85 NA, Olympus) and filter sets for GFP, RFP (tdTomato) and Alexa 568 was used for immunocytochemistry. Z-series images were obtained using 3 μm z-steps at a 1024 × 1024-pixel resolution scanning at 12.5 μs per pixel. The imaging parameters for the red and green channel were kept constant throughout all experiments. Fiji/ImageJ (Schindelin et al. 2012) was used to generate Z-projections and to overlay channels.

### Normalization

Input strength was defined according to the formula:

$$ \mathrm{Input}\ \mathrm{strength}=\frac{\mathrm{EPSC}\ \mathrm{slope}-\mathrm{baseline}\ \mathrm{EPSC}\ \mathrm{slope}}{\left(\mathrm{EPSC}\ \mathrm{slope}+\mathrm{baseline}\ \mathrm{EPSC}\ \mathrm{slope}\right)/2} $$

where (Fig. 2) EPSC slope is the average from 20 to 25 min after oSTDP pairing and baseline the average EPSC slope (~5 min) before pairing, or (Figs 4 and 5) “baseline” is the average EPSC slope of all NT (NT-CA1) neurons from the same slice. This normalization strategy was used as EPSC populations are not normally distributed but follow a log normal distribution. Additionally, this normalization produces symmetrical LTP/LTD windows with the maximum LTP of +2 and maximum LTD of −2 rather than the highly LTP-skewed data produced by dividing solely by (small) baseline values.

### Statistical analysis

GraphPad Prism 9 was used for statistical analysis and to generate plots. All data are represented as mean ± standard error of the mean (SEM). Statistical significance was assumed for *P* < 0.05. For details of the statistical analysis, see Supplementary Table S1.

## Results

### Independent optogenetic control of pre- and post-synaptic action potentials

To independently spike pre- and post-synaptic neurons, we used the red light sensitive ChrimsonR (Klapoetke et al. 2014) and the blue/violet light-sensitive CheRiff (Hochbaum et al. 2014). ChrimsonR-tdTomato was expressed in a small subset of CA3 pyramidal neurons by local AAV injection and several CA1 neurons were electroporated with DNA encoding CheRiff-eGFP (Fig. 1A). Based on calibration experiments (Fig. 1B and C), we selected 405 nm (1 mW mm^−2^) to activate CheRiff-CA1 neurons without activating ChrimsonR-CA3 neurons, and 625 nm (8 mW mm^−2^) to activate ChrimsonR-CA3 neurons. These intensities reliably and selectively evoked single action potentials/spikes in transduced CA3 or CA1 pyramidal neurons using 1–2 ms flashes from 7 to 10 days after transduction (e.g. Fig. 1D, Supplementary Fig. S1). As CheRiff-CA1 neurons were completely insensitive to wavelengths longer than 565 nm, we coexpressed the red fluorescent protein mKate2 to visualize them without spiking the postsynaptic neurons.

### Optogenetic induction of spike-timing-dependent plasticity

As proof of principle, we induced STDP all-optically while patched on the postsynaptic neuron (Fig. 2A and B). We recorded excitatory postsynaptic currents (EPSCs) in CheRiff- or NT CA1 (NT-CA1) neurons while optogenetically stimulating the ChrimsonR-CA3 neurons through the condenser with light flashes at 20 s intervals (594 nm). After collecting baseline responses for 5 min, we switched from voltage- to current-clamp mode. Single presynaptic ChrimsonR-CA3 spikes (300 flashes at 5 Hz) evoked EPSPs that preceded or followed 3-spike bursts by about 10 ms in the transfected CheRiff-CA1 postsynaptic neurons (Tr, 3 flashes at 50 Hz, Fig. 2C). Causal pairing (pre- before post-) induced tLTP (*n* = 12 experiments; *P* = 0.005, paired *t*-test, Fig. 2D and F, for details of statistical analyses see Supplementary Table S1) whereas anticausal pairing (post- before pre-) induced tLTD (*n* = 11 experiments, *P* = 0.02, paired *t*-test, Fig. 2E and F). The normalized changes in synaptic strength after causal and anticausal pairing were significantly different (Fig. 2F, *P* = 0.0003, ANOVA-Sidak). There was no correlation with of post-STDP input strength with baseline EPSC slope (Supplementary Fig. S2) Importantly, there was no change in synapses onto NT-CA1 neurons (*n* = 6 experiments, *P* = 0.9, paired *t*-test, Fig. 2F), indicating that oSTDP was specific to synapses between coactivated CA3 and CA1 neurons and no plasticity was induced by the 5 Hz presynaptic stimulation alone. Thus, using light to induce spike-burst STDP produces an asymmetric tLTD–tLTP window at Schaffer collateral synapses (Bi and Poo 1998; Debanne et al. 1998; Nevian and Sakmann 2006; Wittenberg and Wang 2006; Holbro et al. 2010; Edelmann et al. 2015; Tigaret et al. 2016).

### Input strength is potentiated three days after no-patch oSTDP

**Fig. 3:**
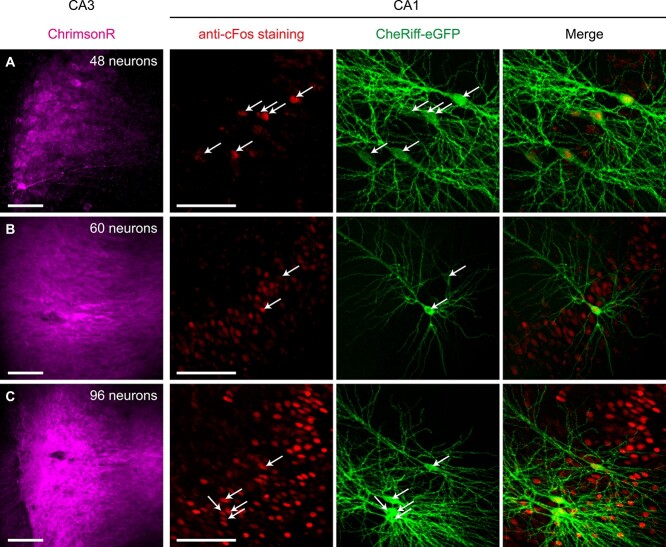
After causal pairing, cFos expression was confined to CheRiff-CA1 neurons when less than 60 ChrimsonR-CA3 neurons were stimulated. Confocal images (average intensity projection) of CA3 and CA1 areas of 3 slice cultures expressing ChrimsonR-tdT in CA3 (magenta) and CheRiff-eGFP in CA1 neurons (green, white arrows indicate nuclei). In red is cFos immunofluorescence. Slices were fixed 1 h after causal pairing stimulation (pattern in Fig. 2D). (A) A slice with fewer than 50 ChrimsonR-CA3 expressing neurons stimulated during pairing. Note that cFos immunoreactivity is restricted to the CheRiff-CA1 neurons and neighboring CA1 neurons are not cFos positive (5 of 6 are cFos positive). (B, C) Slices with more than 50 ChrimsonR-CA3 neurons. Note the cFos positive CA1 neurons in addition to the CheRiff-CA1 neurons. Scale bars 100 μm.

To induce STDP inside the incubator, we constructed illumination towers containing independently controlled, collimated red (625 nm) and violet (405 nm) high-power LEDs to stimulate ChrimsonR-CA3 and CheRiff-CA1 neurons with light pulses of defined intensity (Fig. 1). After causal pairing, we assessed expression of the immediate early gene cFos, which is upregulated in burst-spiking neurons (Schoenenberger et al. 2009) and in neurons that have undergone LTP (for review, see Jaworski et al. 2018). Eighty-three percent of CheRiff-CA1 neurons expressed cFos, indicating they were spiking in bursts during causal pairing as expected (7 slices: 30/36 neurons; e.g. Fig. 3A). Whether NT CA1 neurons also expressed cFos depended critically on the number of ChrimsonR-CA3 neurons being stimulated in the slice. In 5 slices with 36–53 ChrimsonR-CA3 neurons, no NT-CA1 neurons expressed cFos after causal pairing (Fig. 3A). In 2 slices with 60–61 ChrimsonR-CA3 neurons, some NT-CA1 neurons showed cFos staining, and in 2 slices with 96 or more ChrimsonR-CA3 neurons, cFos staining was evident throughout the entire CA1, i.e. the input from CA3, was strong enough to directly drive spiking throughout the CA1 during causal pairing (Fig. 3B and C). As a synapsin promoter drove ChrimsonR expression, local CA3 interneurons were also transduced leading to tight control of spiking in the ChrimsonR-CA3 neurons (Supplementary Fig. S1). Likewise, we did not observe cFos expression in ChrimsonR-CA3 neurons regardless of the number transduced *except* when we stimulated them to fire spike-bursts with both excitatory and inhibitory transmission blocked (Supplementary Fig. S3). Thus, cFos distinguishes cells firing at 5 Hz from burst-firing neurons. Therefore, to avoid driving spikes in neighboring CA1 neurons and restrict induction of STDP to only the synapses between the ChrimsonR-CA3 and CheRiff-CA1 neurons, we adjusted the amount of ChrimsonR virus injected to transduce ~30 CA3 neurons per slice culture (e.g. Fig. 1A).

**Fig. 4:**
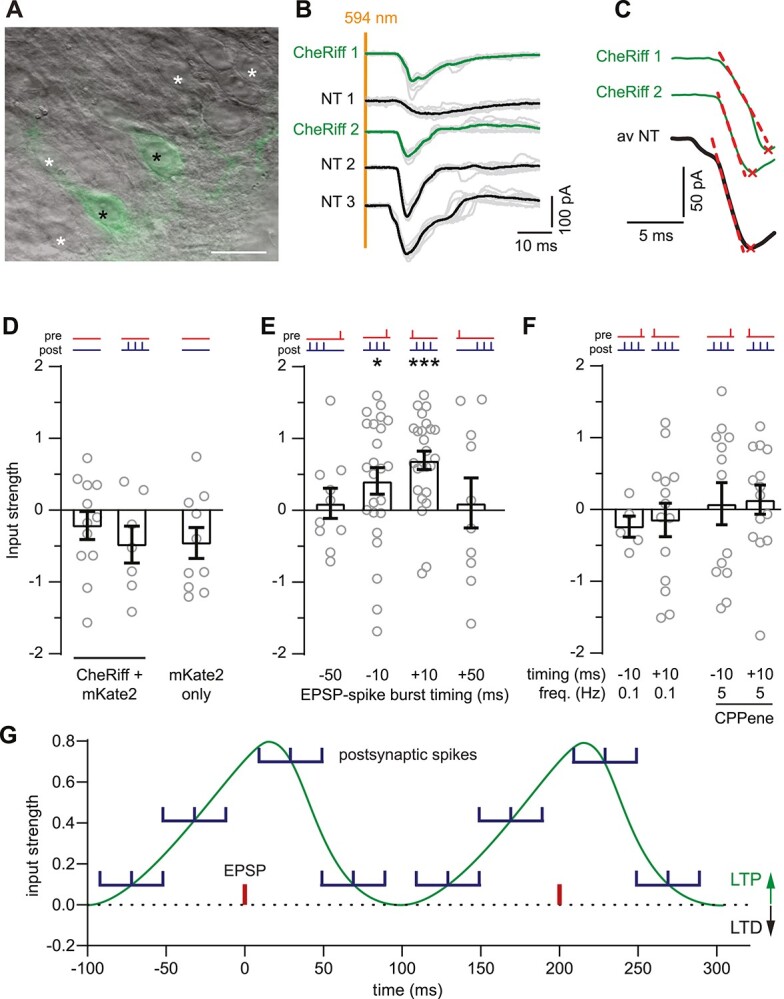
Input to Cheriff-CA1 neurons 3 days after optogenetic STDP. (A) Dodt-contrast image (40x objective) of the CA1 region with overlaid epifluorescence image. Black asterisks: CheRiff-eGFP expressing CA1 pyramidal neurons; white asterisks: NT CA1 pyramidal neurons suitable for recording. Scale bar 25 μm. (B) Yellow light (1 ms, 594 nm) on ChrimsonR-CA3 neurons EPSCs in CA1 neurons of a control (no oSTDP pairing) slice. EPSCs are recorded sequentially from CheRiff-CA1 pyramidal neurons (green, average of 10 gray individual EPSCs) and at least 3 NT CA1 neurons (black average of 10 gray individual EPSCs). (C) Automatically detected EPSC peak (red x) and slope (dashed red line, 20–60% peak) from individual CheRiff-CA1 neurons and the average of NT neurons. (D–F) Red and violet ticks indicate pre- (red) and post-synaptic (violet) light stimulation. (D) Normalized input strength of CheRiff-CA1 neurons recorded from nonpaired control (left: no light stimulation; right: postsynaptic stimulation only) slices and mKate2-CA1 neurons 3 days later. mean ± SEM. *n* = 12; 7; 10 (left to right). (E) Normalized input strength of CheRiff-CA1 neurons 3 days after 300 pairings of single presynaptic and 3 postsynaptic spikes at 5 Hz. During anticausal pairing the last postsynaptic spike occurred −50 or − 10 ms before the EPSP. During causal pairing the first postsynaptic spike occurred +10 or + 50 ms after the EPSP. *n* = 10; 24; 25; 10 (left to right). ^*^P < 0.05, ^***^P < 0.001. (F) Same as (E), but the pairing frequency was reduced to 0.1 Hz (360 pairings in 1 h) or the NMDA receptor antagonist CPPene (1 μM) was in the culture medium (±10 ms pairing 300x at 5 Hz. *n* = 5; 14; 14; 13, left to right). (G) Mean input strength (data from E) as a function of timing between EPSPs (red) and postsynaptic spike bursts (violet, at mean) at 5 Hz repetition frequency. Two complete cycles are illustrated.

Following 2-color light stimulation inside the incubator, the organotypic cultures remained untouched for 3 days. After this incubation period, they were placed in the recording chamber of the patch-clamp setup (Fig. 2A). To determine whether oSTDP had induced long-term changes in synaptic strength, EPSCs were recorded from CA1 neurons in response to stimulating ChrimsonR-CA3 neurons with 1 ms flashes of 594 nm light (Fig. 4A). In these and all following experiments, the experimenter was blind to the stimulation pattern, and stimulated (or otherwise treated) slice cultures were always interspersed with each other and nonpaired controls. To obtain a relative measure of synaptic input strength, we compared the EPSC measured in each transfected CA1 neuron to the average of EPSCs recorded from several NT CA1 neurons in the same field of view (Fig. 4B and C, see methods). There were no differences in input strength of ChrimsonR-CA3 to CheRiff-CA1 neurons in control nonpaired slices (no light, postsynaptic only) or slices with mKate2-only CA1 neurons (Fig. 4D, *P* = 0.62 ANOVA). Thus, expression of the optogenetic tools did not affect synaptic connections, and we pooled the nonpaired control experiments for statistical analysis (see below, Supplementary Table S1). Input strength from ChrimsonR-CA3 onto CheRiff-CA1 neurons was significantly potentiated relative to the input onto nontransduced CA1 neurons 3 days after causal pairing (Fig. 4E, +10 ms: *P* = 0.003, Dunnett’s). Unexpectedly, potentiation (rather than depression) was also the outcome 3 days after anticausal pairing (Fig. 4E, −10 ms: *P* = 0.014, Dunnett’s). Still, the time interval between EPSPs and postsynaptic spikes was important, as input strength did not change when Δ*t* was 50 ms (Fig. 4E, +50 ms: *P* = 0.49, Dunnett’s; −50 ms: *P* = 0.49, Dunnett’s). Thus, 3 days after oSTDP, a clear trace of the 5 Hz induction episode remained if CA3 and CA1 cells were activated synchronously (+/− 10 ms), but not when activated asynchronously (+/− 50 ms).

It has been reported that tLTP is associated with an immediate increase in postsynaptic excitability (Frick et al. 2004; Debanne and Poo 2010; Debanne et al. 2019). We tested for such changes three days after oSTDP, but found no differences in passive electrical parameters or excitability (number of action potentials in response to current injection) between the CheRiff-CA1 and the NT CA1 neurons (Supplementary Figs S4 and S5). Thus, late tLTP reflects a change in synaptic strength rather than altered postsynaptic excitability.

### Late tLTP is frequency and NMDA receptor-dependent

We further investigated the requirements for induction of late tLTP. Previous studies have reported a strong dependence on pairing frequency (Wittenberg and Wang  2006). Similarly, we observed that reducing the pairing frequency from 5 to 0.1 Hz prevented late tLTP in our experiments (0.1 Hz, −10 ms: *P* = 1; +10 ms: *P* = 0.87, Dunnett’s, Fig. 4F). Furthermore, as for classical STDP (Bi and Poo 1998; Debanne et al. 1998; Nevian and Sakmann 2006; Edelmann et al. 2015; Tigaret et al. 2016), late tLTP was abolished when NMDA receptors were blocked during pairing (CPPene *P* = 0.23, Dunnett’s, Fig. 4F).

### The late appearance of tLTP after anticausal pairing is activity-dependent

**Fig. 5:**
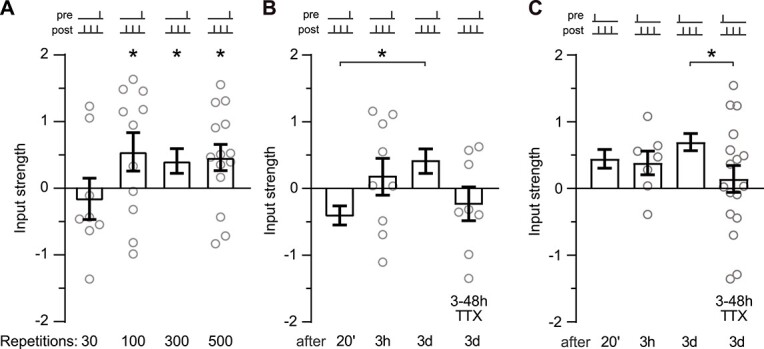
Late tLTP after anticausal pairing is time and activity dependent. (A) Input strength 3 days after anticausal (−10 ms) pairing using different numbers of repetitions *n* = 8; 11; 24; 14 (left to right). The 300 repetition group is replotted from Figure 4E. ^*^P < 0.05 mean ± SEM. (B) Input strength at varying times after anticausal pairing and when spiking was blocked with TTX (1 μM) from 3 to 4 h until 1 day before assessment. *n* = 11, 9, 24, 8 (left to right). ^*^P < 0.05 mean ± SEM The mean ± SEM 20 min and 3 day data are replotted without the individual points from Figures 2F and 4E. (C) Input strength at varying times after causal pairing and when spiking was blocked with TTX (1 μM) from 3 to 4 h until 1 day before assessment. *n* = 12, 7, 25, 17 (left to right). ^*^P < 0.05 mean ± SEM The mean ± SEM 20 min and 3 day data are replotted from Figures 2F and 4E.

While optogenetic tLTD was observed 20 min after induction in patch-clamped neurons, we instead observed tLTP 3 days after in-incubator anticausal oSTDP induction (Fig. 4G). We tested whether late tLTD might be uncovered by changing the number of anticausal pairings (Wittenberg and Wang 2006). However, 100–500 pairings induced potentiation, while 30 repetitions were too few (30 rep.: *P* = 0.98; 100 rep.: *P* = 0.03; 300 rep.: *P* = 0.02; 500 rep.: *P* = 0.03, Dunnett’s, Fig. 5A). Could it be that STDP switches sign with time? We examined the outcome 2–3 h after in-incubator anticausal oSTDP. At this intermediate time point there was no significant change in input strength (Fig. 5B). We reasoned that after oSTDP, circuits containing paired synapses might develop higher levels of spontaneous spiking, increasing reactivation of the synapses between the paired CA3 and CA1 neurons thus leading to tLTP after anticausal pairing (Sadowski et al. 2016; Debanne et al. 2019). Accordingly, we globally suppressed activity starting 3–4 h after anticausal (−10 ms) pairing for 2 days and recorded from the treated slices 3 days later. Late tLTP after anticausal pairing was abolished by this treatment (−10 ms, TTX: *P* = 1, Dunnett’s, Fig. 5B). These results suggest that the synaptic memory of a short episode of coincident activity is actively modified in the circuit.

## Discussion

Previously, the requirement of impaling the postsynaptic neuron with a sharp electrode or using whole-cell or perforated patch recordings to induce STDP has made it impossible to assess the late (i.e. 3 days later) effects of STDP. In the present study, we demonstrate: (1) that the opsins CheRiff and ChrimsonR can be used to independently spike CA3 and CA1 neurons, (2) that theta frequency optogenetic pairing in causal sequence (first CA3, then CA1) induces tLTP, whereas anticausal pairing induces tLTD during patch-clamp recordings, and (3) that the long-term consequence of oSTDP is a symmetric potentiation-only window at short timing intervals (±10 ms) and no potentiation at longer intervals (±50 ms). Thus, we have directly demonstrated something long assumed to be true, that information about the coincidence of paired spiking is stored for an extended period in the strength of the connections between those specific neurons. As we normalized the strength of inputs onto the paired postsynaptic neurons to the input onto neighboring (unpaired) neurons, global changes during the 3-day incubation period would not affect our results. Similarly, increasing expression of presynaptic ChrimsonR would equally affect EPSC slopes in paired and neighboring NT neurons. Underlying late tLTP may be strengthening of existing synapses, an increase in the number of synapses between the paired neurons, or a combination of both. We cannot at present distinguish between these possibilities: Our method includes 2 different fluorescent labels in pre- and post-synaptic neurons, but to unequivocally identify and count synapses with light microscopy, additional functional information is required, e.g. activity-dependent labeling (Perez-Alvarez et al. 2020).

The protocol we selected, a single presynaptic spike paired with a burst of 3 spikes at 50 Hz, was demonstrated in neocortex to induce robust tLTP or tLTD with causal (+10 ms) or anticausal (−10 ms) timing (Nevian and Sakmann 2006). In hippocampal CA1 neurons, spike-burst pairing is also effective or even necessary for STDP induction (Meredith et al. 2003; e.g. Wittenberg and Wang 2006; Frey et al. 2009; Edelmann et al. 2015). Since during our short-term patch-clamp recordings, oSTDP reproduced asymmetric STDP with tLTD and tLTP after anticausal and causal pairing, respectively (Markram 1997; Bi and Poo 1998; Debanne et al. 1998; Meredith et al. 2003; Zhang et al. 2009), we surmise that optically and electrically induced spiking are not fundamentally different. As is well established for electrode-induced STDP (Nevian and Sakmann 2006), late tLTP depends on NMDA receptors, strongly suggesting that elevated intracellular [Ca^2+^] in the postsynaptic neuron is essential. A further similarity of late oSTDP to electrode-induced STDP is the strong frequency-dependence (Wittenberg and Wang 2006): At a pairing repetition rate of 5 Hz, late tLTP is observed, but not when the pairing rate is reduced to 0.1 Hz. As in previous STDP studies, there is no potentiation at spike intervals of ±50 ms. When postsynaptic spikes occur about 50 ms after EPSPs, GABA_A_ currents from feed-forward inhibition are strongly active in CA1 neurons (Samulack and Lacaille 1993) and may reduce postsynaptic calcium influx, as has been demonstrated for back-propagating action potentials (Marlin and Carter 2014). Even with inhibition blocked, single spine synaptic calcium transients are reduced if paired bAPs are delayed by 40 ms compared with shorter delays (Holbro et al. 2010). Postsynaptic AMPA currents have decayed and no longer contribute to the bAP-induced relief of NMDAR Mg^2+^ block. Likewise, postsynaptic spikes that precede EPSPs by more than a few ms will not contribute to the single-spine Ca^2+^ transients.

As optical anticausal pairing induced tLTD during patch-clamp recordings, we were very surprised by the lack of late tLTD 3 days after oSTDP induction. While we cannot exclude that somewhere in the vast parameter space a protocol for late tLTD may exist, none of the modified protocols we tested (increasing or decreasing the number of anticausal pairing repetitions, changing the pairing interval, decreasing the pairing frequency) reduced synaptic input three days after induction. Nor is tLTD apparent at the earliest time window (1–3 hours) feasible after noninvasive oSTDP induction. Ideally, we would have assessed plasticity in the incubator-stimulated slices even sooner but this would require simultaneously patching 4 neurons within 20 min of stimulation. Of note, we included the calcium chelator 0.2 mM EGTA in our internal solution, which could affect synaptic plasticity. However, others report similar bidirectional STDP using either comparable EGTA concentrations (e.g. Bi and Poo 1998; Nevian and Sakmann 2006; Campanac and Debanne 2008; Tigaret et al. 2016) or no EGTA (e.g. Meredith et al. 2003; Kampa et al. 2006; Nevian and Sakmann 2006; Wittenberg and Wang 2006; Edelmann et al. 2015). Interestingly, Pang et al. (2019) who used extracellular electrodes to induce and record STDP, failed to observe LTD at early (<30 min) time points. During their 4-h long recordings, LTP persisted for timings from −10 to +20 ms and LTD slowly appeared only for the anticausal pairing interval of −20 ms. Both their study and the present work suggest that STDP has hitherto unappreciated slow dynamics that can include a change in sign.

When we disrupted spontaneous activity in the slice cultures 3 h after oSTDP, the “synaptic memory” of oSTDP was erased. We speculate that the endogenous activity in the circuit becomes biased towards “replaying” the optogenetically induced sequences, reactivating the paired synapses. Interestingly, maintenance and consolidation of memories in vivo also requires ongoing activity and NMDA receptors (Shimizu et al. 2000; Cui et al. 2004). Transient knock-out of NMDA receptors wipes out previously encoded memory, but does not prevent the future acquisition of new memories (Cui et al. 2004). Indeed, the notion that memory consolidation requires replay, for instance during sleep, is well supported (Wilson and McNaughton 1994; Squire et al. 2015). Directly testing this speculative hypothesis will require continuous monitoring of circuit activity with synaptic resolution, ideally simultaneously at many synapses over several days.

That the early effects of oSTDP replicated the classical asymmetric STDP window strongly argues against optogenetic artifacts, such as calcium entry through the opsins, being responsible for the difference in early and late effects. As channelrhodopsins are distributed in the plasma membrane, perhaps optical generation of spiking is closer to being physiological than the standard method of inducing spikes by somatic current injection, both of which are of course artificial manipulations: Natural spikes arise from summation of active excitatory synapses distributed over the dendritic arbor, not from depolarization of the soma. In the postsynaptic neuron, dendritic depolarization is likely more important than spiking per se for synaptic plasticity (Lisman and Spruston 2005; Kumar and Mehta 2011). Pairing mossy fiber stimulation with CA3–CA3 synaptic inputs induces tLTP or tLTD of the CA3–CA3 synapses without spiking of the postsynaptic CA3 neuron (Brandalise and Gerber 2014). Likewise, at CA3–CA1 synapses, tLTP can occur without postsynaptic spikes (Hardie and Spruston 2009). Therefore, if CheRiff-CA1 cells occasionally missed spiking and the dendrites only depolarized in response to the violet light flashes, synaptic plasticity could still be expected.

While the majority of STDP studies report both potentiation and depression, “LTP-only” STDP windows have been observed at human hippocampal, human and rat neocortical synapses (Testa-Silva et al. 2010; Brzosko et al. 2015) and in the mouse hippocampus (Wittenberg and Wang 2006), most commonly in the presence of increased dopamine (Zhang et al. 2009; Brzosko et al. 2015). If presynaptic activity is paired with prolonged postsynaptic bursts (plateau potentials), the timing window for CA3–CA1 potentiation can be extended to several seconds in the causal and anticausal direction (Bittner et al. 2017). While there are many possible explanations for these discrepant outcomes of early oSTDP, we speculate that if the neurons are left intact and postsynaptic strength is assessed after several days, the outcome of repeated coincident activity at CA3–CA1 synapses may always be potentiation at short pairing intervals. What we do not believe is that there is no LTD. Heterosynaptic LTD or generalized downscaling processes are important mechanisms to conserve total synaptic weight and prevent runaway potentiation (Turrigiano 2017). Although unlikely, we cannot exclude that the nonpaired synapses onto the NT-CA1 neurons and the ±50 ms paired synapses underwent LTD relative to the ±10 ms paired synapses, as we assessed relative synaptic strength. To put the relationship between synaptic plasticity and memory storage onto a firm empirical basis, we need to investigate how neuronal activity modifies synaptic connections though STDP-like processes over extended periods of time.

## Supplementary Material

anisimova_2021_cerebal_cortex_supplementary_material_resubmission2_bhac050

## References

[ref1] Adamantidis AR, Zhang F, Aravanis AM, Deisseroth K, de Lecea L. Neural substrates of awakening probed with optogenetic control of hypocretin neurons. Nature. 2007:450:420–424. 17943086 10.1038/nature06310 PMC6744371

[ref2] Bi G, Poo M. Synaptic modifications in cultured hippocampal neurons: dependence on spike timing, synaptic strength, and postsynaptic cell type. J Neurosci. 1998:18:10464–10472. 9852584 10.1523/JNEUROSCI.18-24-10464.1998 PMC6793365

[ref3] Bing Z, Meschede C, Chen G, Knoll A, Huang K. Indirect and direct training of spiking neural networks for end-to-end control of a lane-keeping vehicle. Neural Netw. 2020:121:21–36. 31526952 10.1016/j.neunet.2019.05.019

[ref4] Bittner KC, Milstein AD, Grienberger C, Romani S, Magee JC. Behavioral time scale synaptic plasticity underlies CA1 place fields. Science (80-). 2017:357:1033–1036. 10.1126/science.aan3846 PMC7289271 28883072

[ref5] Boyden ES, Zhang F, Bamberg E, Nagel G, Deisseroth K. Millisecond-timescale, genetically targeted optical control of neural activity. Nat Neurosci. 2005:8:1263–1268. 16116447 10.1038/nn1525

[ref6] Brandalise F, Gerber U. Mossy fiber-evoked subthreshold responses induce timing-dependent plasticity at hippocampal CA3 recurrent synapses. Proc Natl Acad Sci. 2014:111:4303–4308. 24550458 10.1073/pnas.1317667111 PMC3964078

[ref7] Brzosko Z, Schultz W, Paulsen O. Retroactive modulation of spike timing-dependent plasticity by dopamine. elife. 2015:4:e09685. 26516682 10.7554/eLife.09685 PMC4626806

[ref8] Brzosko Z, Mierau SB, Paulsen O. Neuromodulation of spike-timing-dependent plasticity: past, present, and future. Neuron. 2019:103:563–581. 31437453 10.1016/j.neuron.2019.05.041

[ref9] Campanac E, Debanne D. Spike timing-dependent plasticity: a learning rule for dendritic integration in rat CA1 pyramidal neurons. J Physiol. 2008:586:779–793. 18048448 10.1113/jphysiol.2007.147017 PMC2254206

[ref10] Costa RP, Froemke RC, Sjöström PJ, van Rossum MCW. Unified pre- and post-synaptic long-term plasticity enables reliable and flexible learning. elife. 2015:4:e09457. 26308579 10.7554/eLife.09457 PMC4584257

[ref11] Cui Z, Wang H, Tan Y, Zaia KA, Zhang S, Tsien JZ. Inducible and reversible NR1 knockout reveals crucial role of the NMDA receptor in preserving remote memories in the brain. Neuron. 2004:41:781–793. 15003177 10.1016/s0896-6273(04)00072-8

[ref12] Debanne D, Poo M-M. Spike-timing dependent plasticity beyond synapse - pre- and post-synaptic plasticity of intrinsic neuronal excitability. Front Synaptic Neurosci. 2010:2:21. 21423507 10.3389/fnsyn.2010.00021 PMC3059692

[ref13] Debanne D, Gähwiler BH, Thompson SM. Long-term synaptic plasticity between pairs of individual CA3 pyramidal cells in rat hippocampal slice cultures. J Physiol. 1998:507:237–247. 9490845 10.1111/j.1469-7793.1998.237bu.x PMC2230782

[ref14] Debanne D, Inglebert Y, Russier M. Plasticity of intrinsic neuronal excitability. Curr Opin Neurobiol. 2019:54:73–82. 30243042 10.1016/j.conb.2018.09.001

[ref15] Edelmann E, Cepeda-Prado E, Franck M, Lichtenecker P, Brigadski T, Leßmann V. Theta burst firing recruits BDNF release and signaling in postsynaptic CA1 neurons in spike-timing-dependent LTP. Neuron. 2015:86:1041–1054. 25959732 10.1016/j.neuron.2015.04.007

[ref16] Foncelle A, Mendes A, Jędrzejewska-Szmek J, Valtcheva S, Berry H, Blackwell KT, Venance L. Modulation of spike-timing dependent plasticity: towards the inclusion of a third factor in computational models. Front Comput Neurosci. 2018:12:49. 30018546 10.3389/fncom.2018.00049 PMC6037788

[ref17] Frey MC, Sprengel R, Nevian T. Activity pattern-dependent long-term potentiation in neocortex and hippocampus of GluA1 (GluR-A) subunit-deficient mice. J Neurosci. 2009:29:5587–5596. 19403825 10.1523/JNEUROSCI.5314-08.2009 PMC6665861

[ref18] Frick A, Magee J, Johnston D. LTP is accompanied by an enhanced local excitability of pyramidal neuron dendrites. Nat Neurosci. 2004:7:126–135. 14730307 10.1038/nn1178

[ref19] Gee CE, Ohmert I, Wiegert JS, Oertner TG. Preparation of slice cultures from rodent hippocampus. Cold Spring Harb Protoc. 2017:2017:126–130. 10.1101/pdb.prot094888 28148854

[ref20] Gerstner W, Kempter R, van Hemmen JL, Wagner H. A neuronal learning rule for sub-millisecond temporal coding. Nature. 1996:383:76–81. 8779718 10.1038/383076a0

[ref21] Hardie J, Spruston N. Synaptic depolarization is more effective than back-propagating action potentials during induction of associative long-term potentiation in hippocampal pyramidal neurons. J Neurosci. 2009:29:3233–3241. 19279260 10.1523/JNEUROSCI.6000-08.2009 PMC2826107

[ref22] Hochbaum DR, Zhao Y, Farhi SL, Klapoetke N, Werley CA, Kapoor V, Zou P, Kralj JM, Maclaurin D, Smedemark-Margulies N, et al. All-optical electrophysiology in mammalian neurons using engineered microbial rhodopsins. Nat Methods. 2014:11:825–833. 24952910 10.1038/nmeth.3000 PMC4117813

[ref23] Holbro N, Grunditz A, Wiegert JS, Oertner TG. AMPA receptors gate spine Ca(2+) transients and spike-timing-dependent potentiation. Proc Natl Acad Sci. 2010:107:15975–15980. 20798031 10.1073/pnas.1004562107 PMC2936625

[ref24] Huber D, Petreanu L, Ghitani N, Ranade S, Hromádka T, Mainen Z, Svoboda K. Sparse optical microstimulation in barrel cortex drives learned behaviour in freely moving mice. Nature. 2008:451:61–64. 18094685 10.1038/nature06445 PMC3425380

[ref25] Jaworski J, Kalita K, Knapska E. c-Fos and neuronal plasticity: the aftermath of Kaczmarek’s theory. Acta Neurobiol Exp (Wars). 2018:78:287–296. 30624427

[ref26] Kampa BM, Letzkus JJ, Stuart GJ. Requirement of dendritic calcium spikes for induction of spike-timing-dependent synaptic plasticity. J Physiol. 2006:574:283–290. 16675489 10.1113/jphysiol.2006.111062 PMC1817800

[ref27] Kheradpisheh SR, Ganjtabesh M, Thorpe SJ, Masquelier T. STDP-based spiking deep convolutional neural networks for object recognition. Neural Netw. 2018:99:56–67. 29328958 10.1016/j.neunet.2017.12.005

[ref28] Klapoetke NC, Murata Y, Kim SS, Pulver SR, Birdsey-Benson A, Cho YK, Morimoto TK, Chuong AS, Carpenter EJ, Tian Z, et al. Independent optical excitation of distinct neural populations. Nat Methods. 2014:11:338–346. 24509633 10.1038/nmeth.2836 PMC3943671

[ref29] Kumar A, Mehta MR. Frequency-dependent changes in NMDAR-dependent synaptic plasticity. Front Comput Neurosci. 2011:5:38. 21994493 10.3389/fncom.2011.00038 PMC3182344

[ref30] Lisman J, Spruston N. Postsynaptic depolarization requirements for LTP and LTD: a critique of spike timing-dependent plasticity. Nat Neurosci. 2005:8:839–841. 16136666 10.1038/nn0705-839

[ref31] Markram H . Regulation of synaptic efficacy by coincidence of postsynaptic APs and EPSPs. Science (80-). 1997:275:213–215. 10.1126/science.275.5297.213 8985014

[ref32] Marlin JJ, Carter AG. GABA-A receptor inhibition of local calcium signaling in spines and dendrites. J Neurosci. 2014:34:15898–15911. 25429132 10.1523/JNEUROSCI.0869-13.2014 PMC4244464

[ref33] Masquelier T, Guyonneau R, Thorpe SJ. Competitive STDP-based spike pattern learning. Neural Comput. 2009:21:1259–1276. 19718815 10.1162/neco.2008.06-08-804

[ref34] Meredith RM, Floyer-Lea AM, Paulsen O. Maturation of long-term potentiation induction rules in rodent hippocampus: role of GABAergic inhibition. J Neurosci. 2003:23:11142–11146. 14657173 10.1523/JNEUROSCI.23-35-11142.2003 PMC6741060

[ref36] Nagel G, Szellas T, Huhn W, Kateriya S, Adeishvili N, Berthold P, Ollig D, Hegemann P, Bamberg E. Channelrhodopsin-2, a directly light-gated cation-selective membrane channel. Proc Natl Acad Sci. 2003:100:13940–13945. 14615590 10.1073/pnas.1936192100 PMC283525

[ref37] Nevian T, Sakmann B. Spine Ca2+ signaling in spike-timing-dependent plasticity. J Neurosci. 2006:26:11001–11013. 17065442 10.1523/JNEUROSCI.1749-06.2006 PMC6674669

[ref38] Pang KKL, Sharma M, Krishna-K. K, Behnisch T, Sajikumar S. Long-term population spike-timing-dependent plasticity promotes synaptic tagging but not cross-tagging in rat hippocampal area CA1. Proc Natl Acad Sci. 2019:116:5737–5746. 30819889 10.1073/pnas.1817643116 PMC6431168

[ref35] Perez-Alvarez A, Fearey BC, O'Toole RJ, Yang W, Arganda-Carreras I, Lamothe-Molina PJ, Moeyaert B, Mohr MA, Panzera LC, et al. Freeze-frame imaging of synaptic activity using SynTagMA. Nat Commun. 2020:1:2464. 10.1038/s41467-020-16315-4 PMC7235013 32424147

[ref39] Sadowski JHLP, Jones MW, Mellor JR. Sharp-wave ripples orchestrate the induction of synaptic plasticity during reactivation of place cell firing patterns in the hippocampus. Cell Rep. 2016:14:1916–1929. 26904941 10.1016/j.celrep.2016.01.061 PMC4785795

[ref40] Samulack DD, Lacaille J-C. Hyperpolarizing synaptic potentials evoked in CA1 pyramidal cells by glutamate stimulation of interneurons from the oriens/alveus border of rat hippocampal slices. II. Sensitivity to GABA antagonists. Hippocampus. 1993:3:345–358. 8102583 10.1002/hipo.450030309

[ref41] Schindelin J, Arganda-Carreras I, Frise E, Kaynig V, Longair M, Pietzsch T, Preibisch S, Rueden C, Saalfeld S, Schmid B, et al. Fiji: an open-source platform for biological-image analysis. Nat Methods. 2012:9:676–682. 22743772 10.1038/nmeth.2019 PMC3855844

[ref42] Schoenenberger P, Gerosa D, Oertner TG. Temporal control of immediate early gene induction by light. PLoS One. 2009:4:e8185. 19997631 10.1371/journal.pone.0008185 PMC2780714

[ref43] Serrano-Gotarredona T, Masquelier T, Prodromakis T, Indiveri G, Linares-Barranco B. STDP and STDP variations with memristors for spiking neuromorphic learning systems. Front Neurosci. 2013:7:2. 23423540 10.3389/fnins.2013.00002 PMC3575074

[ref44] Shimizu E, Tang YP, Rampon C, Tsien JZ. NMDA receptor-dependent synaptic reinforcement as a crucial process for memory consolidation. Science (80-). 2000:290:1170–1174. 10.1126/science.290.5494.1170 11073458

[ref45] Song S, Miller KD, Abbott LF. Competitive Hebbian learning through spike-timing-dependent synaptic plasticity. Nat Neurosci. 2000:3:919–926. 10966623 10.1038/78829

[ref46] Squire LR, Genzel L, Wixted JT, Morris RG. Memory consolidation. Cold Spring Harb Perspect Biol. 2015:7:a021766. 26238360 10.1101/cshperspect.a021766 PMC4526749

[ref47] Stoppini L, Buchs PA, Muller D. A simple method for organotypic cultures of nervous tissue. J Neurosci Methods. 1991:37:173–182. 1715499 10.1016/0165-0270(91)90128-m

[ref48] Suter BA, O’Connor T, Iyer V, Petreanu LT, Hooks BM, Kiritani T, Svoboda K, Shepherd GMG. Ephus: multipurpose data acquisition software for neuroscience experiments. Front Neural Circuits. 2010:4:100. 21960959 10.3389/fncir.2010.00100 PMC3176413

[ref49] Testa-Silva G, Verhoog MB, Goriounova NB, Loebel A, Hjorth J, Baayen JC, De Kock CPJ, Mansvelder HD. Human synapses show a wide temporal window for spike-timing-dependent plasticity. Front Synaptic Neurosci. 2010:2:12. 21423498 10.3389/fnsyn.2010.00012 PMC3059666

[ref50] Tigaret CM, Olivo V, Sadowski JHLP, Ashby MC, Mellor JR. Coordinated activation of distinct Ca2+ sources and metabotropic glutamate receptors encodes Hebbian synaptic plasticity. Nat Commun. 2016:7:10289. 26758963 10.1038/ncomms10289 PMC4735496

[ref51] Turrigiano GG . The dialectic of Hebb and homeostasis. Philos Trans R Soc B Biol Sci. 2017:372:20160258. 10.1098/rstb.2016.0258 PMC5247594 28093556

[ref52] Wiegert JS, Gee CE, Oertner TG. Viral vector-based transduction of slice cultures. Cold Spring Harb Protoc. 2017a:2017a:131–134. 10.1101/pdb.prot094896 28148855

[ref53] Wiegert JS, Gee CE, Oertner TG. Single-cell electroporation of neurons. Cold Spring Harb Protoc. 2017b:2017b:135–138. 10.1101/pdb.prot094904 28148856

[ref54] Wilson MA, McNaughton BL. Reactivation of hippocampal ensemble memories during sleep. Science. 1994:265:676–679. 8036517 10.1126/science.8036517

[ref55] Wittenberg GM, Wang SS. Malleability of spike-timing-dependent plasticity at the CA3-CA1 synapse. J Neurosci. 2006:26:6610–6617. 16775149 10.1523/JNEUROSCI.5388-05.2006 PMC6674029

[ref56] Zhang J-C, Lau P-M, Bi G-Q. Gain in sensitivity and loss in temporal contrast of STDP by dopaminergic modulation at hippocampal synapses. Proc Natl Acad Sci U S A. 2009:106:13028–13033. 19620735 10.1073/pnas.0900546106 PMC2713390

