# Supplementary material for: Spike-timing-dependent plasticity rewards synchrony rather than causality
Source: Cereb Cortex. 2022 Feb 24;33(1):23–34. doi: 10.1093/cercor/bhac050 (PMC9758582; doi:10.1093/cercor/bhac050)
Supplement: anisimova_2021_cerebal_cortex_supplementary_material_resubmission2_bhac050 [file anisimova_2021_cerebal_cortex_supplementary_material_resubmission2_bhac050.docx]

# Supplemental Information


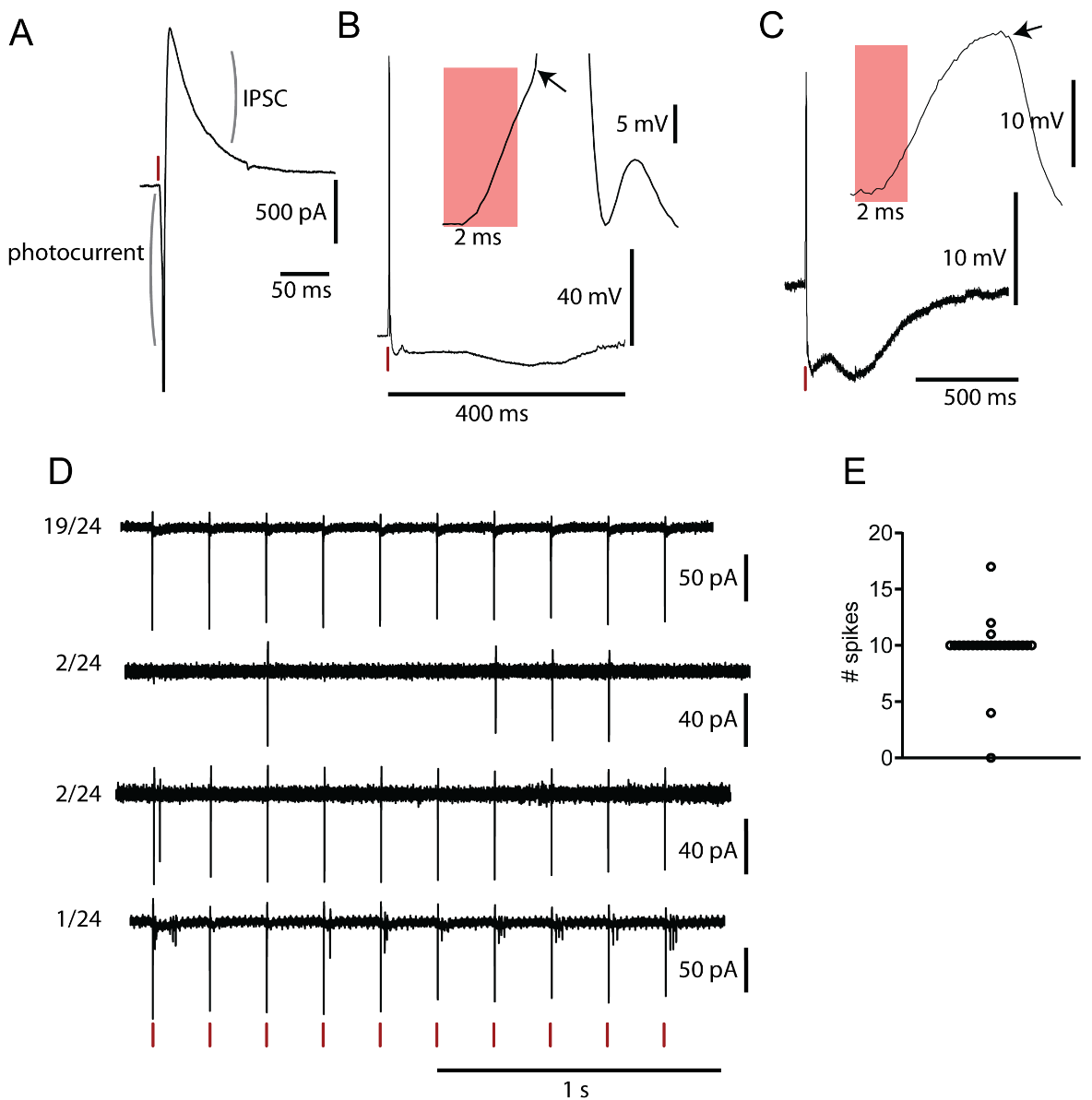


**Supplementary Figure 1. CA3-ChrimsonR cell responses to 2 ms, 625 nm red light flashes at t = 600 ms with intact synaptic transmission.**  rAAV2/10 synapsin-ChrimsonR-tdTomato was locally injected into CA3. **A,** A ChrimsonR-CA3 cell voltage-clamped at -54.4 mV. The light intensity was at spike threshold for this neuron (determined in cell-attached mode, 2.8 mW mm^-2^). Note the outward inhibitory postsynaptic current immediately following the truncated inward photo- and action current. **B,** Same neuron stimulated with light supra-threshold for this neuron in current clamp (10 mW mm^-2^). A single action potential is elicited followed by a pronounced hyperpolarization. Note, the light (red shading) is off *before* the action potential is initiated (arrow in inset, time 2.5 ms after start of light flash). **C,** Response of same neuron to light stimulation sub-threshold for this neuron (2.5 mW mm^-2^). Note the photoresponse is truncated by an inhibitory postsynaptic potential 5 ms after start of the light flash (arrow in inset). **D,** Example cell-attached recordings of ChrimsonR-CA3 neurons in medium in response to 10 x 2 ms 8 mW mm^-2^ 625 nm at 5 Hz. 19 of 24 neurons fired exactly 10 spikes, 2 of 24 less than 10, 2 of 24 1-2 extra spikes and 1 of 24 may have fired small bursts of spikes. **E,** quantification of number of spikes fired from 24 cell-attached recordings as in D.

**
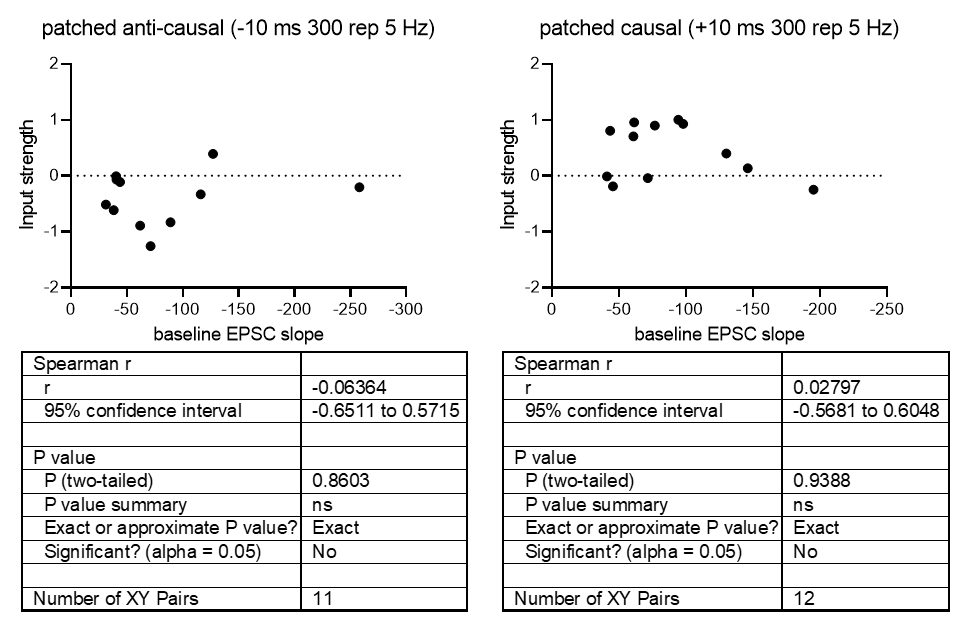
**

**Supplementary Figure 2.** **The slope of baseline EPSCs does not correlate with the sign or magnitude of the post oSTDP input strength onto CheRiff-CA1 neurons.** The average slope of baseline EPSCs vs. input strength 20-25 min. after oSTDP from experiments in Figure 2.

$$Input strength= \frac{EPSC slope -baseline EPSC slope}{(EPSC slope+baseline EPSC slope)/2}$$

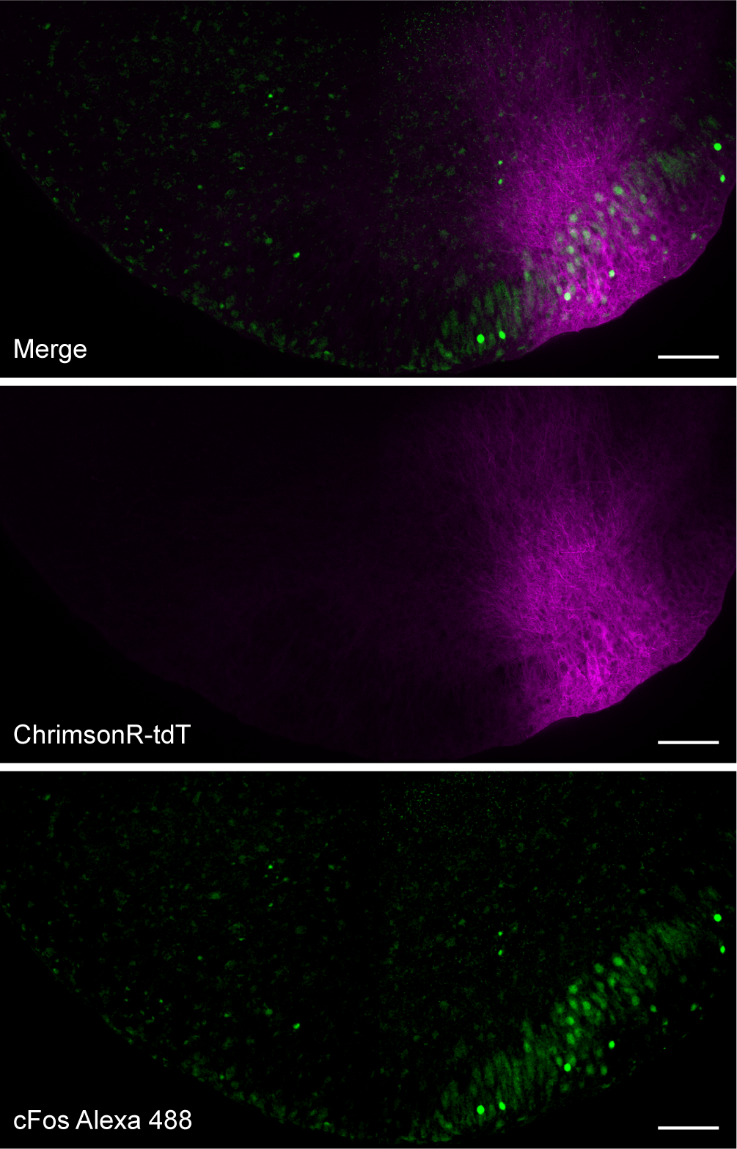


**Supplementary Figure 3. Burst-firing is necessary for cFos expression in ChrimsonR-CA3 neurons.** Confocal images of CA3 area of a hippocampal slice culture fixed 90 minutes after burst stimulation (synaptic transmission blocked with: 1 µM CPPene, 100 µM Picrotoxin, 10 µM NBQX; red light: 3 flashes at 50 Hz repeated 300x at 5 Hz, 625 nm, 8 mW mm^-2^). The ChrimsonR-tdTomato channel (magenta) is a minimum-intensity projection and a median-intensity projection was used for anti-cFos immunostaining (green). Approximately half of the burst-stimulated ChrimsonR-CA3 neurons were cFos-positive as were several non-transduced neighboring CA3 neurons. Scale bars 100 μm.

#
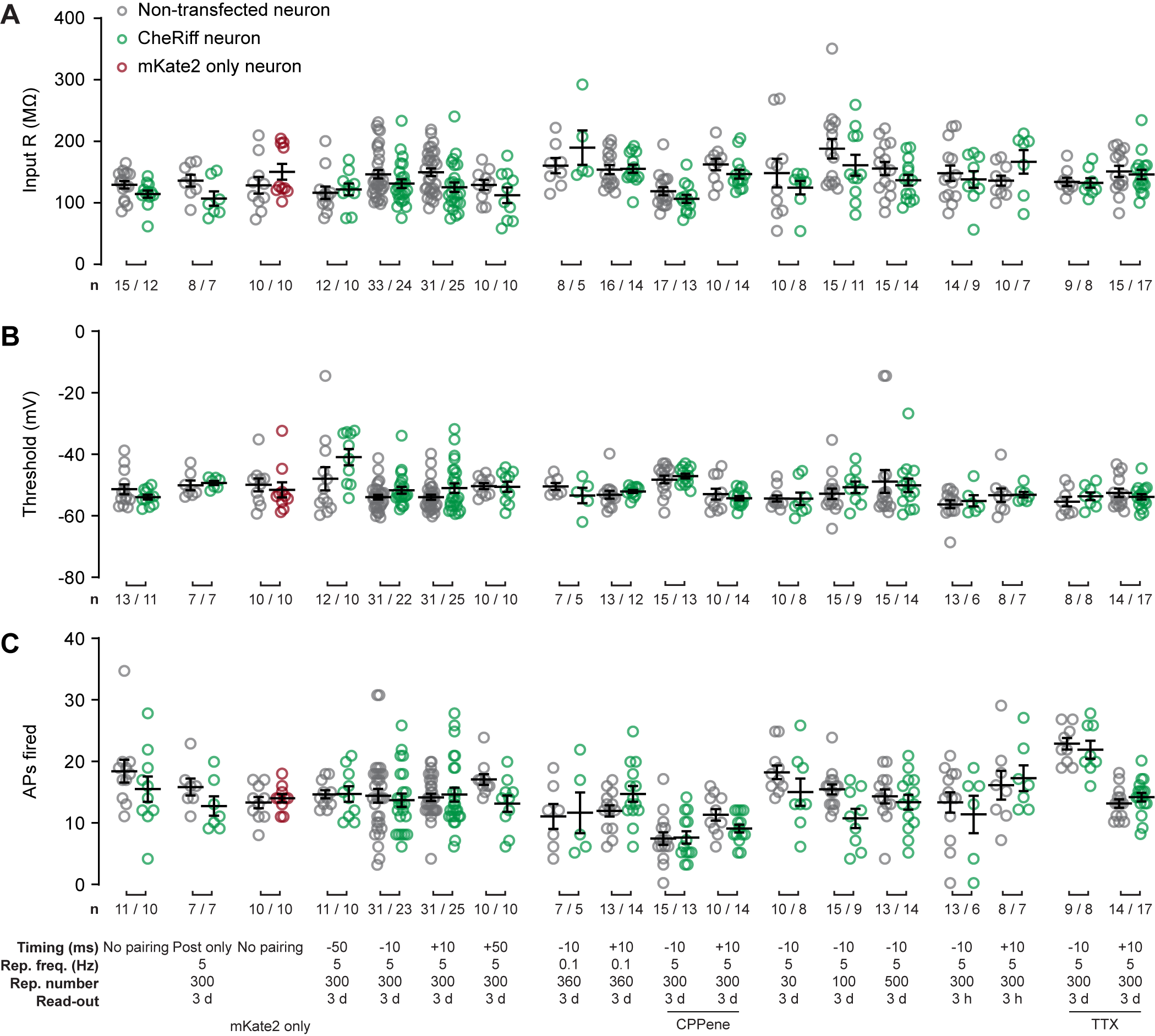


**Supplementary Figure 4. Comparison of passive and active cell parameters of CheRiff-CA1 and neighboring NT-CA1 neurons from oSTDP experiments in Figure 4. A,** Input resistance of all NT-CA1 and CheRiff-CA1 neurons. **B,** Action potential threshold. **C,** Numbers of action potentials fired in response to a 400 pA current step. There were no significant differences between NT and CheRiff-CA1 neurons in any of the treatment groups (Kolmogorov-Smirnov). CPPene: 1 µM CPPene during oSTDP; TTX: 1 µM tetrodotoxin 4-48 h after oSTDP. n number of NT-CA1 / CheRiff-CA1 neurons. Plotted are individual data points, mean ± SEM. As the current clamp measurements (**B** and **C**) were performed last, they were missed in some recordings.

**
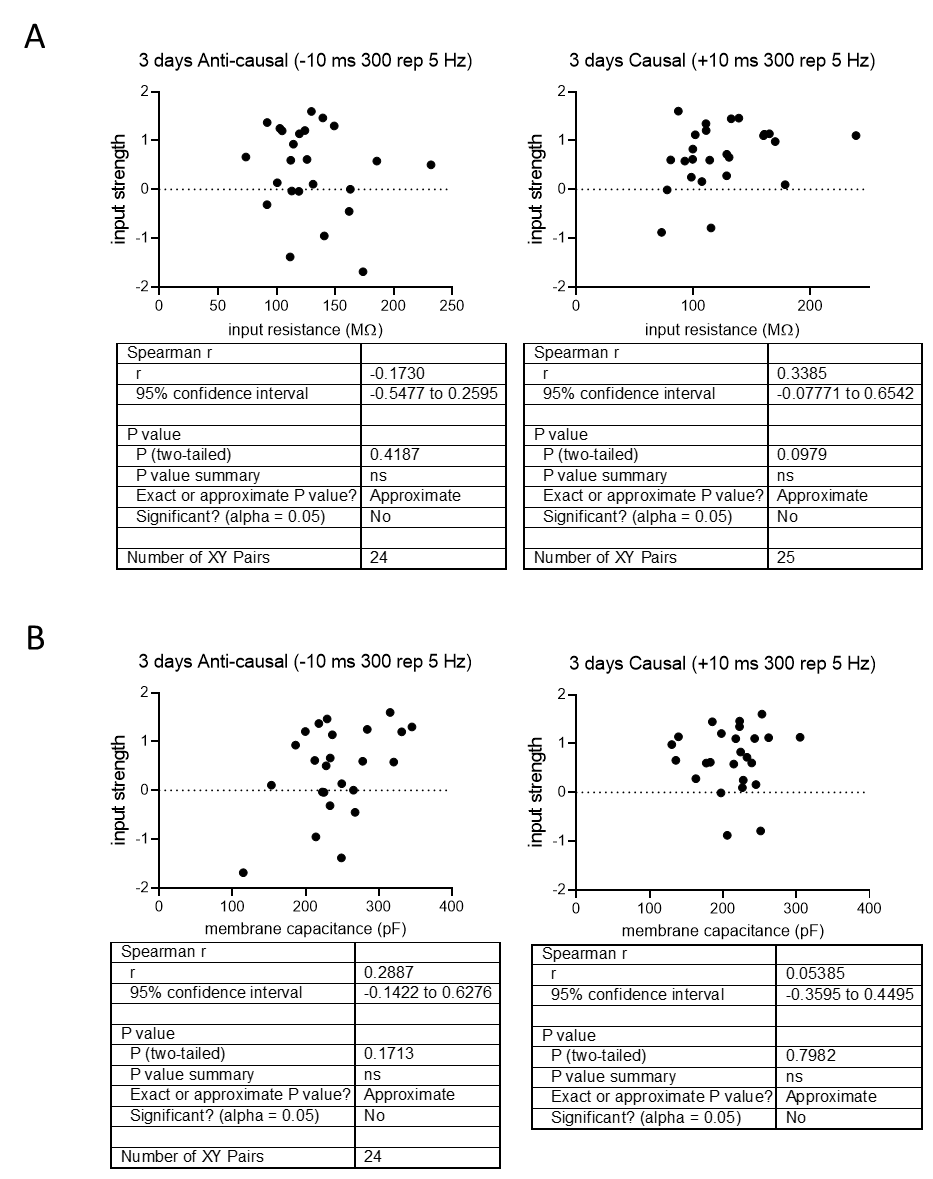
**

**Supplementary Figure 5.** **Example correlations of cellular parameters with input strength after oSTDP.** **A,** Input resistance of the CheRiff-CA1 neurons vs input strength after oSTDP. **B,** Membrane capacitance of the CheRiff-CA1 neurons vs input strength after oSTDP.

| Figure | | Test | |  | P value | Comment |
| --- | --- | --- | --- | --- | --- | --- |
| 2 | D | Kolmogorov-Smirnov | | D = 1.0 | <0.0001 | Before vs after |
|  | E | Kolmogorov-Smirnov | | D = 0.92 | 0.0003 | Before vs after |
|  | In text | Ratio paired t-tests  before vs after pairing | | n = 11, t = 2.748, df = 10 | 0.020 | Anti-causal |
|  | In text |  |  | n = 12, t = 3.473, df = 11 | 0.0052 | Causal |
|  | In text |  |  | n = 6, t = 0.1249, df = 5 | 0.91 | NT |
|  | F | one-way ANOVA | | F (2, 26) = 10.69 | 0.0004 | 3 groups |
|  |  | Šídák's multiple comparisons test | | | 0.0003 | Anti-causal vs. Causal |
| 4 | D | one-way ANOVA | | F(2,26) = 0.49 | 0.62 | 3 groups |
|  |  | Šídák's multiple comparisons test | | | 0.80 | No light vs post only |
|  |  |  |  |  | 0.79 | No light vs mKate2 |
|  |  |  |  |  | 1 | Post only vs mKate2 |
|  | E | one-way ANOVA | | F (4, 83) = 4.842 | 0.0015 | No pairing + 4 groups |
|  |  | Dunnett's multiple comparisons vs no pairing | | | 0.49 | -50 ms 3d 5Hz |
|  |  |  |  |  | 0.014 | -10 ms 3d 5Hz |
|  |  |  |  |  | 0.0003 | +10 ms 3d 5Hz |
|  |  |  |  |  | 0.49 | +50 ms 3d 5Hz |
|  | F | one-way ANOVA | | F (4, 60) = 0,8354 | 0.51 | No pairing + 4 groups |
|  |  | Dunnett's multiple comparisons vs no pairing | | | 0.99 | -10ms 0.1 Hz |
|  |  |  |  |  | 0.95 | +10 ms 0.1 Hz |
|  |  |  |  |  | 0.51 | -10 ms CPPene |
|  |  |  |  |  | 0.36 | +10 ms CPPene |
| 5 | A | one-way ANOVA | F (4, 71) = 3,434 | | 0.013 | No pairing + 4 groups |
|  |  | Dunnett's multiple comparisons vs no pairing | | | 0.98 | No pairing vs 30 rep |
|  |  |  |  |  | 0.028 | No pairing vs 100 rep |
|  |  |  |  |  | 0.021 | No pairing vs 300 rep |
|  |  |  |  |  | 0.035 | No pairing vs 500 rep |
|  | B | one-way ANOVA | F (3,60) = 2.41 | | 0.031 | 4 groups |
|  |  | Dunnett's multiple comparisons vs 20’ -10 ms | | | 0.25 | 3h -10 ms |
|  |  |  |  |  | 0.018 | 3d -10 ms |
|  |  |  |  |  | 0.9 | 3d -10 ms TTX (3-48h) |
|  | C | one-way ANOVA | F (3,48) = 1.254 | | 0.031 | 4 groups |
|  |  | Dunnett's multiple comparisons vs 3d +10 ms | | | 0.61 | 20’ +10 ms |
|  |  |  |  |  | 0.59 | 3h +10 ms |
|  |  |  |  |  | 0.029 | 3d +10 ms TTX |

**Supplementary Table 1: Summary of statistical analyses** All analyses were performed using GraphPad Prism v8. P values are adjusted for multiple comparisons.
